# Plumbagin Enhances the Anticancer Effects of PF Chemotherapy via Downregulation of the PI3K/AKT/mTOR/p70S6K Pathway in Human Tongue Squamous Cell Carcinoma

**DOI:** 10.1155/2023/8306514

**Published:** 2023-02-13

**Authors:** Shu-Ting Pan, Fang-Fei Ye, Gan Huang, Jia-Xuan Qiu

**Affiliations:** Department of Oral and Maxillofacial Surgery, The First Affiliated Hospital of Nanchang University, Nanchang, Jiangxi 330006, China

## Abstract

Cisplatin plus 5-fluorouracil (PF) is used as the standard neoadjuvant chemotherapy (also called preoperative chemotherapy) in the treatment of tongue squamous cell carcinoma (TSCC). Although PF chemotherapy reduces the distant metastasis of TSCC, the five-year survival rate has not significantly improved. In recent years, components considered in traditional Chinese medicine have been researched as adjuvant drugs for radiotherapy and chemotherapy. Plumbagin (PB) is a quinone component isolated from *Plumbago zeylanica* L. Notably, PB demonstrates numerous anticancer properties. In order to examine the chemosensitization effect of PB on PF and its associated mechanisms, *in vitro* experiments using TSCC Cal27 and cisplatin (CDDP)-resistant Cal27/CDDP cells were carried out in the present study, and the results were subsequently verified using nude mice xenografts. Results of the present study demonstrated that PB enhanced the anticancer effects of PF on the proliferation, migration, and invasion of Cal27 and Cal27/CDDP cells. Cell cycle assays demonstrated that both Cal27 and Cal27/CDDP cells were arrested in the S phase following the combined treatment of PF and PB. Moreover, the PF and PB combination group induced higher levels of apoptosis in Cal27 and Cal27/CDDP cells compared with the group treated with PF alone. In addition, the results of the present study demonstrated that combined PB and PF inhibited the PI3K/AKT/mTOR/p70S6K pathway in TSCC cells. Moreover, the weight and volumes of tumors in nude mice were reduced following treatment with a combination of PF and PB. Results of the present study also demonstrated that the expression levels of Ki67 were markedly reduced in the combined treatment group compared with the group treated with PF alone. In summary, the results of the present study demonstrated that PB enhanced the PF sensitivity of TSCC through induction of S-phase arrest and apoptosis via the PI3K/AKT/mTOR/p70S6K pathway.

## 1. Introduction

Tongue squamous cell carcinoma (TSCC) is the most common malignancy of the head and neck region, with a high rate of regional lymph node metastasis and high morbidity and mortality [1, 2]. Notably, the five-year survival rate of TSCC is between 48.0 and 70.9%, and the incidence of recurrence following curative treatment of TSCC is 26.5–51.0% [3, 4]. Neoadjuvant chemotherapy, as a key method for the treatment of TSCC, plays an important role in controlling tumor size and preserving oral function [5]. In the neoadjuvant chemotherapy regimen for TSCC, cisplatin plus 5-fluorouracil (PF) is the standard option. Although neoadjuvant chemotherapy reduces distant metastases in patients with TSCC, five-year survival rates have not been significantly improved [6, 7]. The main factors affecting efficacy are the decreased sensitivity of tumor cells to chemotherapeutic drugs and the emergence of chemoresistance [8]. Therefore, the discovery of novel methods to improve the sensitivity of TSCC to PF chemotherapy is required.

In recent years, an increasing number of researchers have focused on treatment options derived from natural plants [9, 10]. Plumbagin (PB) is a quinone component isolated from the rhizome of *Plumbago zeylanica* L., which exerts antioxidant, anti-inflammatory, antibacterial, anticancer, and other biological activities [11]. *In vitro and in vivo* experiments demonstrated that PB exhibits antitumor properties in various cancers, such as breast cancer, pancreatic cancer, and lung cancer, with no notable toxicity observed in healthy cells [12]. Notably, PB demonstrates antiproliferative [13], antimetastatic [14], proapoptotic [15], and proautophagic cell death [16] effects in both tumor cells and animal models. Results of our previous studies demonstrated that PB might induce reactive oxygen species (ROS) and apoptosis in TSCC cells by targeting multiple signaling proteins including MAPKs and AKT/mTOR [17, 18]. However, the sensitization effect of PB on chemotherapeutic drugs and the associated mechanism are yet to be fully elucidated.

The present study aimed to explore the sensitization role and mechanisms of PB on PF chemotherapy, using the Cal27 and cisplatin-resistant Cal27/CDDP TSCC cell lines. We found that PB enhanced the anticancer effects of PF on the proliferation, migration, and invasion of Cal27 and Cal27/CDDP cells. *In vivo* study revealed that PB and PF in combination significantly inhibited TSCC xenograft tumor growth.

## 2. Materials and Methods

### 2.1. Chemicals and Reagents

PB was purchased from Merck KgaA (Sigma-Aldrich). Fetal bovine serum (FBS) and 5-fluorouracil were purchased from Gemini Co. and Xudong Haipu pharmaceutical Co., respectively. Cisplatin was bought from Qilu pharmaceutical Co. DMEM, DMSO, CCK-8 kit, Giemsa, Matrigel, Hoechst 33342, phosphate buffer saline (PBS), bovine serum albumin (BSA), RIPA buffer, protease inhibitor cocktail, BCA protein assay kit, and PVDF were all purchased from Solarbio Science and Technology Co., Ltd (Beijing, China). EDU kits were purchased from Guangzhou RiboBio Co., Ltd. Cell cycle kits were bought from Lianke Biological Technology Co. AnnexinV-FITC/propidium iodide apoptosis detection kits were purchased from Beibo Co. Primary antibodies against Bcl-2, Bcl-xL, Bax, Bad, PI3K, AKT, phosphorylated (p)-AKT, mTOR, p-mTOR, p70S6K, and p-p70S6K were purchased from Cell Signaling Technology (Danvers, MA, USA). Primary antibody against Ki67 and *β*-actin was purchased from Proteintech (Wuhan, China). Rabbit and mouse secondary antibodies (horseradish peroxidase-conjugated antibodies) were purchased from Kangwei Co.

### 2.2. Cell Line and Culture Conditions

TSCC cell lines (Cal27) and TSCC chemoresistant cell lines (Cal27/CDDP) were granted by Sun Yat-sen Memorial Hospital (Guangzhou, China). The chemoresistant cell line Cal27CDDP was created by exposing Cal27 cells to cisplatin at the indicated time [19]. Both of the aforementioned TSCC cells were cultured in DMEM containing 10% (v/v) FBS at 37°C in a humidified incubator with 5% CO_2_.

### 2.3. Cell Viability and Combination Index (CI) Analysis

The CCK-8 test was carried out to examine the effects of different concentrations of PB, and/or 5-Fu, and/or CDDP on cell viability. Cal27 and Cal27/CDDP cells were cultured in 96-well plates for 24 h with a density of 5 × 10^3^. Then, the cells were treated with PB (1.25–20 *μ*M) and/or 5-Fu (160–2560 *μ*g/ml) and/or CDDP (2.5–40 *μ*g/ml) for 24 h to determine the individual and combined effects of PB and PF. Subsequently, 10 *μ*l CCK-8 solution was added to each well, and the plates were incubated for 2 h. The optical density (OD) was measured at 450 nm in a Spark TM microplate reader (Tecan, Inc., Switzerland). Cell viability was calculated as a percentage of the control cells. Cell viability (%) =  (OD treatment-OD blank)/(OD control-OD blank) × 100%. The median inhibitory concentration (IC_50_) was calculated with growth inhibition curves fitted to the data using GraphPad Prism 8.0.

The combined effects of PB and PF on TSCC cells were evaluated using the CI according to the median dose-effect analysis reported by Chou and Talalay [20]. Fractional inhibition (FA) = 1-fraction of surviving cells. The corresponding CI values were analyzed using CompuSyn Software. CI < 1 represents synergistic effect; =1 represents additive cytotoxicity; >1 represents antagonistic effect.

### 2.4. Edu Assay

Cal27 and Cal27/CDDP cells were incubated in 96-well plates with a density of 5 × 10^3^ for 24 h. Then, the cells were treated with 2.5 *μ*M PB or PF (320 *μ*g/ml 5-Fu + 2.5 *μ*g/ml CDDP) alone or PB + PF in combination for another 24 h. The previous medium was removed, and cells were washed with PBS three times. A total of 100 *μ*l Edu solution (50 *μ*M) diluted with culture medium at the ratio of 1000 : 1 was injected into each well and cocultured for 2 h. Subsequently, 4% paraformaldehyde was used to fix the cells, and Hoechst 33342 was added to stain the DNA. Cells were observed by using an inverted fluorescence microscope.

### 2.5. Colony Formation Assay

Cal27 and Cal27/CDDP cells were cultured in six-well plates with 800 cells in each well. 2.5 *μ*M PB or PF (320 *μ*g/ml 5-Fu + 2.5 *μ*g/ml CDDP) alone or PB + PF was employed to treat both cell types for 24 h. Following 2-week incubation, cells were washed with PBS three times and fixed in 4% paraformaldehyde solution for 20 min. Cells were subsequently stained with 2 ml Giemsa solution for 30 min. Colonies with more than 50 cells were scored. Colony formation rate =  (colony number/seeded cell number) ×  100%.

### 2.6. Transwell Invasion Assay

The transwell invasion assay was carried out using 24-well plates inserted with 8 *μ*m chamber coated with Matrigel. In the upper chamber, cells were cultured in the FBS-free medium with a density of 3 × 10^4^. A medium containing 20% FBS was injected into the lower chamber. Cells were subsequently treated with 2.5 *μ*M PB or PF (320 *μ*g/ml 5-Fu + 2.5 *μ*g/ml CDDP) alone or PB + PF for 24 h. Cells were fixed under the membrane and stained with 0.1% crystal violet for 10 min. Cell numbers were counted and photographed with 4 random microscopic fields (×50).

### 2.7. Wound Healing Assay

Cell migration was determined using a wound healing assay. Cells were incubated in 6-well plates (1 × 10^5^ cells/well) with high-glucose medium without FBS until 85% confluence. Cells were scratched from the well surface with a 200 *μ*l pipette. Cells were subsequently treated with 2.5 *μ*M PB or PF (320 *μ*g/ml 5-Fu + 2.5 *μ*g/ml CDDP) alone or PB + PF. After incubation for 24 h, cell migration to the scratches was imaged at 50x magnification.

### 2.8. Cell Cycle Analysis

Cal27 and Cal27/CDDP cells were incubated in 6-well plates with a density of 5 × 10^5^ cells/ml for overnight attachment. Cells were subsequently treated with 2.5 *μ*M PB or PF (320 *μ*g/ml 5-Fu + 2.5 *μ*g/ml CDDP) alone or PB + PF for 24 h. Cells were trypsinized and fixed in ice-cold 75% ethanol overnight. A cell cycle detection kit (Lianke Biological Technology Co., China) was employed to stain the DNA of the cells. After 30 min, 1 × 10^4^ cells were subjected to cell cycle analysis using flow cytometry (Beckman Coulter, Inc.).

### 2.9. Apoptosis Assay

Cal27 and Cal27/CDDP cells were incubated in 6-well plates with a density of 5 × 10^5^ cells/ml for overnight attachment. Cells were treated with 2.5 *μ*M PB or PF (320 *μ*g/ml 5-Fu + 2.5 *μ*g/ml CDDP) alone or PB + PF for 24 h. Cells were then trypsinized and washed three times with cold PBS. AnnexinV-FITC and PI were used to incubate cells for 15 min at room temperature. Thereafter, 200 *μ*l 1 × binding buffer was added to each well, and apoptosis was quantified using flow cytometry.

### 2.10. Western Blot Analysis

Cal27 and Cal27/CDDP cells were cultured in 6-well plates with a density of 5 × 10^5^ cells/ml for overnight attachment. Cells were subsequently treated with 2.5 *μ*M PB or PF (320 *μ*g/ml 5-Fu + 2.5 *μ*g/ml CDDP) alone or PB + PF for 24 h. Cells were rinsed with PBS and lysed with RIPA buffer on ice. Protein concentrations were determined via a BCA protein assay kit. Equal amounts of protein samples were loaded on 5–15% SDS-PAGE gels. Proteins were then transferred to PVDF membranes. Membranes were blocked in 5% BSA at room temperature for 2 h and subsequently probed with primary antibodies against Bcl-2 (1 : 1000 dilution; 15071T), Bcl-xL (1 : 1000 dilution; 2762S), Bax (1 : 1000 dilution; 41162S), Bad (1 : 1000 dilution; 9268S), PI3K (1 : 1000 dilution; 4263S), AKT (1 : 1000 dilution; 4685S), p-AKT (Ser 473) (1 : 1000 dilution; 4060S), mTOR (1 : 1000 dilution; 2972S) p-mTOR (Ser 2448) (1 : 1000 dilution; 5536S), p70S6K(1 : 1000 dilution; 9202S), p-p70S6K (Ser 371) (1 : 1000 dilution; 9208T), and *β*-actin (1 : 5000 dilution; 81115-1-RR) overnight at 4°C. Subsequently, membranes were incubated with secondary antibodies (Mouse CW0102; Rabbit CW0103; 1 : 2000 dilution) for 1 h at room temperature. The membranes were stained using ECL chemiluminescence and captured using a Bio-Rad system (Bio-Rad Laboratories Inc.). Image software (Quantity one V4.5.0) was used to calculate the gray value of each blot.

### 2.11. Tumor Xenograft Model

All male BALB/c nude mice with an average body weight of 20 g (certificate no. SCXK 2016-0002) were obtained from SLAC Jinda Animal Co., Ltd. The animal experiments had been approved by the Ethics Committee of Nanchang University (ethics approval no. SYXK2015-0003). Mice were housed in a standard specific-pathogen-free environment. 0.15 ml cell suspension containing 1 × 10^6^ Cal27/CDDP cells were injected into the right flank of each mouse. Xenograft tumor growth was monitored daily. When the tumor reached ∼80 mm^3^, 24 mice were divided (*n* = 6 in each group) into four groups: (i) the control group, mice were injected with 0.9% saline; (ii) the PB group, mice were injected with 3 mg/kg PB every other day; (iii) the PF group, mice were injected with 4 mg/kg 5-Fu and CDDP every 3 days; and (iv) the PB + PF group, both PB and PF were administrated in combination, in concentrations as previously described. The tumor volumes and body weights of the mice were measured every day. Tumor volume = *a* ×  *b*^2^/2 (a, the largest diameter; *b*, the shortest diameter of the tumor). After 21 days, all mice were sacrificed; tumors were then isolated and weighed. 10% formalin was used to fix the heart, liver, spleen, lung, and kidney. After being dehydrated in ethanol and embedded in paraffin, a series of paraffin sections (5 *μ*m) of the abovementioned organs were stained with hematoxylin-eosin (H and E). All the stained samples were analyzed using a light microscope with 400x magnification.

### 2.12. Immunohistochemistry

All xenograft tumors were excised and fixed in 4% paraformaldehyde for further embedding in paraffin. Subsequently, the paraffin samples were cut into 4 *μ*m slides. The slides were blocked and incubated in Ki67 antibody solution (1 : 2000 dilution; 27309-1-AP). The expression level of Ki67 was examined by counting the number of positive cells from five randomly selected fields with 400x magnification (Olympus BX 53).

### 2.13. Statistical Analysis

All experiments were performed in triplicate, and the data were presented as the mean ± standard deviation. Multiple comparisons were evaluated using a one-way ANOVA and Tukey's post hoc test. When GraphPad 8.0 software was used for statistical analysis, *P* < 0.05 was considered statistically significant.

## 3. Results

### 3.1. PB and PF in Combination Inhibit the Proliferation, Invasion, and Migration of TSCC Cells

**Figure 1:**
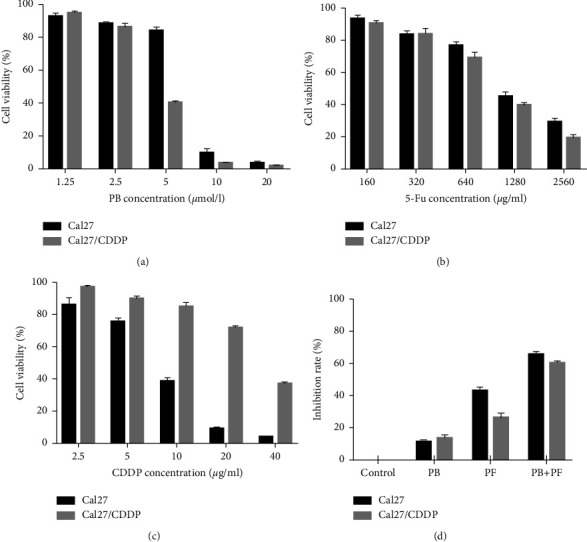
Viability of TSCC cells after various treatments. (a-c) Inhibition of the proliferation of Cal27 and Cal27/CDDP cells following treatment with PB, 5-Fu, and CDDP. (d) A combination of PB and PF exerted synergistic cytotoxic effects in Cal27 and Cal27/CDDP cells. TSCC, tongue squamous cell carcinoma; PB, plumbagin; 5-Fu, 5-fluorouracil; and PF, cisplatin plus 5-fluorouracil.

A CCK-8 assay was carried out to examine the effects of PB (1.25, 2.5, 5, 10, and 20 *μ*M), 5-Fu (160, 320, 640, 1280, and 2560 *μ*g/ml), CDDP (2.5, 5, 10, 20, and 40 *μ*g/ml) alone and in combination on the viability of Cal27 and Cal27/CDDP cells (Figures 1(a)–1(c)). Results of the present study demonstrated that PB, 5-Fu, and CDDP inhibited cell viability in a concentration-dependent manner. In Cal27 cells, the IC50s of PB, 5-Fu, and CDDP were 6.745 *μ*M, 1267 *μ*g/ml, and 7.965 *μ*g/ml, respectively. In Cal27/CDDP cells, the IC50s of PB, 5-Fu, and CDDP were 4.402 *μ*M, 1008 *μ*g/ml, and 31.06 *μ*g/ml, respectively.

**Figure 2:**
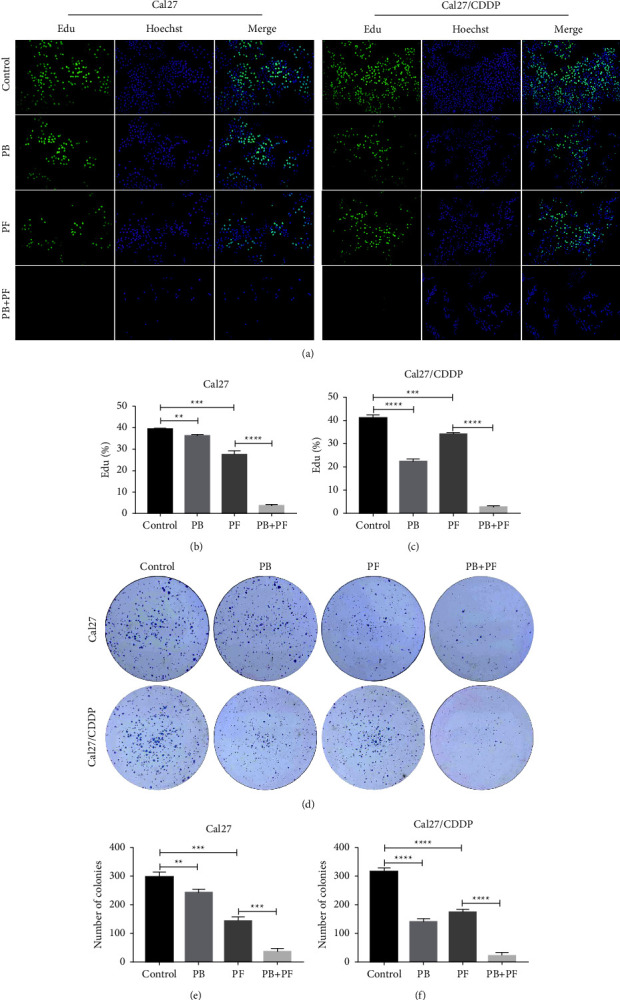
Combined treatment of PB and PF inhibited the proliferation and colony formation of TSCC cells. (a) Results of the Edu assay demonstrated the inhibited proliferation of Cal27 and Cal27/CDDP cells following the treatment with PB and/or PF. (b-c) Histogram of Edu fluorescence in Cal27 and Cal27/CDDP cells. (d) Colony formation of Cal27 and Cal27/CDDP cells following the treatment with PB and/or PF. (e-f) Histogram of numbers of colonies of Cal27 and Cal27/CDDP cells. Independent biological experiments were repeated three times. ^*∗∗*^*P* < 0.01, ^*∗∗∗*^*P* < 0.001 and ^*∗∗∗∗*^*P* < 0.0001. PB, plumbagin; PF, cisplatin plus 5-fluorouracil; and TSCC, tongue squamous cell carcinoma.

**Figure 3:**
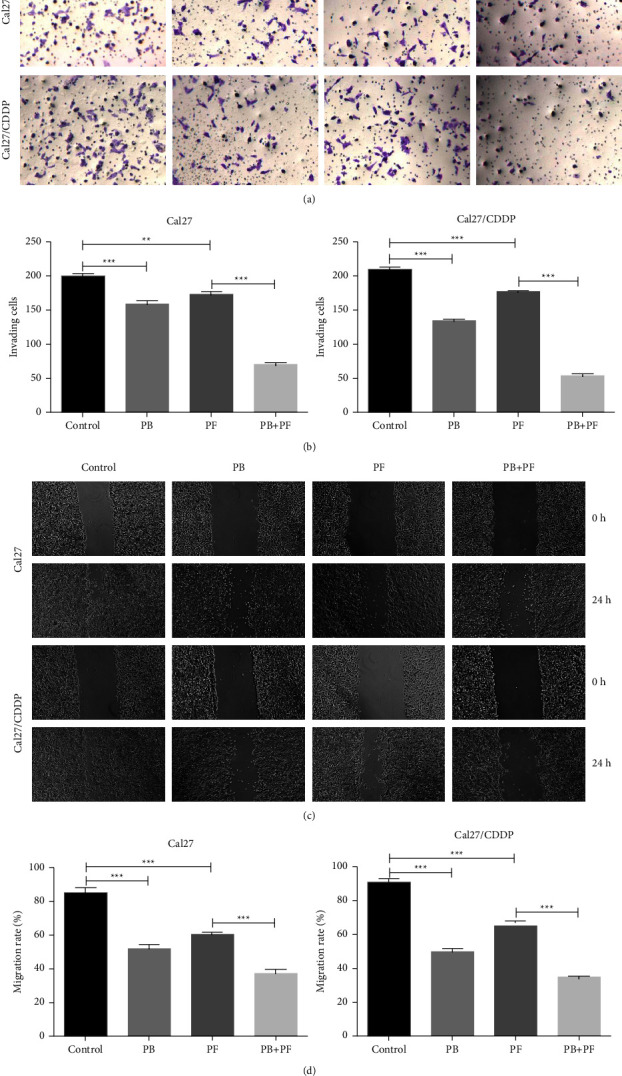
Combined treatment of PB and PF inhibited the migration and invasion of TSCC cells. (a) Results of the transwell assay demonstrated the invasion of Cal27 and Cal27/CDDP cells following treatment with PB and/or PF. (b) Histogram of invading Cal27 and Cal27/CDDP cells. (c) Results of the scratch test demonstrated the migration of Cal27 and Cal27/CDDP cells following treatment with PB and/or PF. (d) Histogram of migration rate of Cal27 and Cal27/CDDP cells. Independent biological experiments were repeated three times (magnification, ×50; scale bar, 200 *μ*m). ^*∗∗*^*P* < 0.01, ^*∗∗∗*^*P* < 0.001. PB, plumbagin; PF, cisplatin plus 5-fluorouracil; and TSCC, tongue squamous cell carcinoma.

Thus, concentrations of 2.5 *μ*M PB, 320 *μ*g/ml 5-Fu, and 2.5 *μ*g/ml CDDP were selected for further experiments. Both Cal27 and Cal27/CDDP cells were divided into four groups, namely, (i) the control group, cells treated with medium only; (ii) PB group, cells treated with 2.5 *μ*M PB; (iii) the PF group, cells treated with 320 *μ*g/ml 5-Fu and 2.5 *μ*g/ml CDDP; and (iv) the PF + PB group, cells treated with both PB and PF, concentrations as previously described. Compared with PF alone, treatment with PB + PF significantly enhanced the inhibition of Cal27 and Cal27/CDDP cells (Figure 1(d)). According to the median dose-effect analysis by Chou and Talalay, the CI values of the combination of PB and PF were 0.73550 and 0.82662 in Cal27 and Cal27/CDDP cells, respectively (Supplementary Table SI). These results demonstrated that the combination of PB and PF exerted synergistic cytotoxic effects in Cal27 and Cal27/CDDP cells. Subsequently, an Edu assay was used to examine the proliferation of TSCC cells. Compared with PF alone, PB + PF treatment markedly decreased the green fluorescence intensity by 87.60% in Cal27 cells and 91.79% in Cal27/CDDP cells (Figures 2(a)–2(c)). A colony formation assay was carried out to examine the colony formation of TSCC cells. Compared with PF alone, PB + PF treatment notably decreased the colonies by 75.06% in Cal27 cells and by 86.42% in Cal27/CDDP cells (Figures 2(d)–2(f)). The transwell assay revealed that PB + PF significantly reduced the invasion ability of Cal27 and Cal27/CDDP cells (Figures 3(a) and 3(b)). The wound healing assay demonstrated that PB + PF significantly decreased the migration ability of Cal 27 and Cal27/CDDP cells, compared with PF alone (Figures 3(c) and 3(d)). These results indicated that PB synergistically enhanced the cytotoxicity of PF in TSCC cells, and the combination of PB and PF inhibited the invasion and migration of TSCC cells.

### 3.2. PB and PF in Combination Promotes S Arrest in TSCC Cells

**Figure 4:**
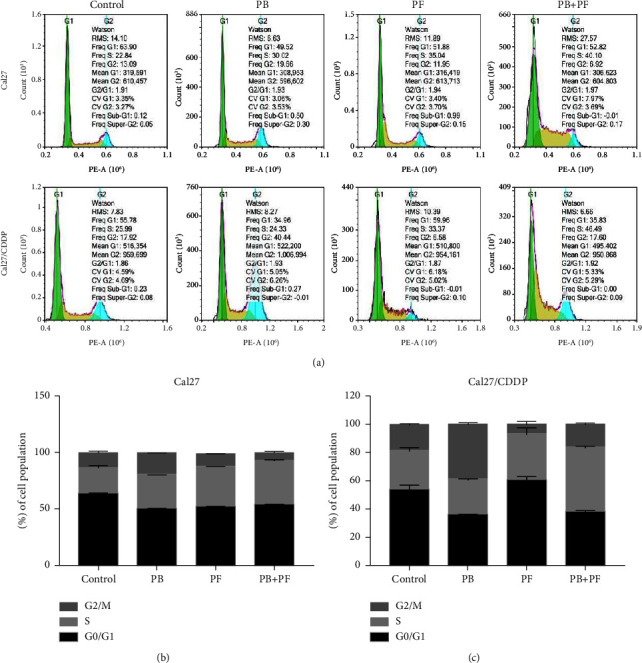
Combined PB and PF induced S phase arrest of TSCC cells. (a) A representative cell cycle in Cal27 and Cal27/CDDP cells following the treatment with PB and/or PF. (b-c) A histogram of cell cycle distribution in Cal27 and Cal27/CDDP cells. PB, plumbagin; PF, cisplatin plus 5-fluorouracil; and TSCC, tongue squamous cell carcinoma.

Flow cytometry was carried out to examine the cell cycle distribution following the drug treatment. Cal27 and Cal27/CDDP cells were treated with 2.5 *μ*M PB, PF (320 *μ*g/ml 5-Fu and 2.5 *μ*g/ml CDDP), and PB + PF for 24 h. In Cal27 cells, compared with PF alone, PB + PF significantly increased the S phase by 1.31-fold (Figures 4(a) and 4(b)). In Cal27/CDDP cells, compared with the PF single group, the PB and PF combination group significantly increased the S phase by 3.67-fold (Figure 4(c)). This indicated that combined PB and PF could significantly induce S phase arrest in TSCC cells.

### 3.3. PB Enhances the Proapoptosis Effect of PF in TSCC Cells

**Figure 5:**
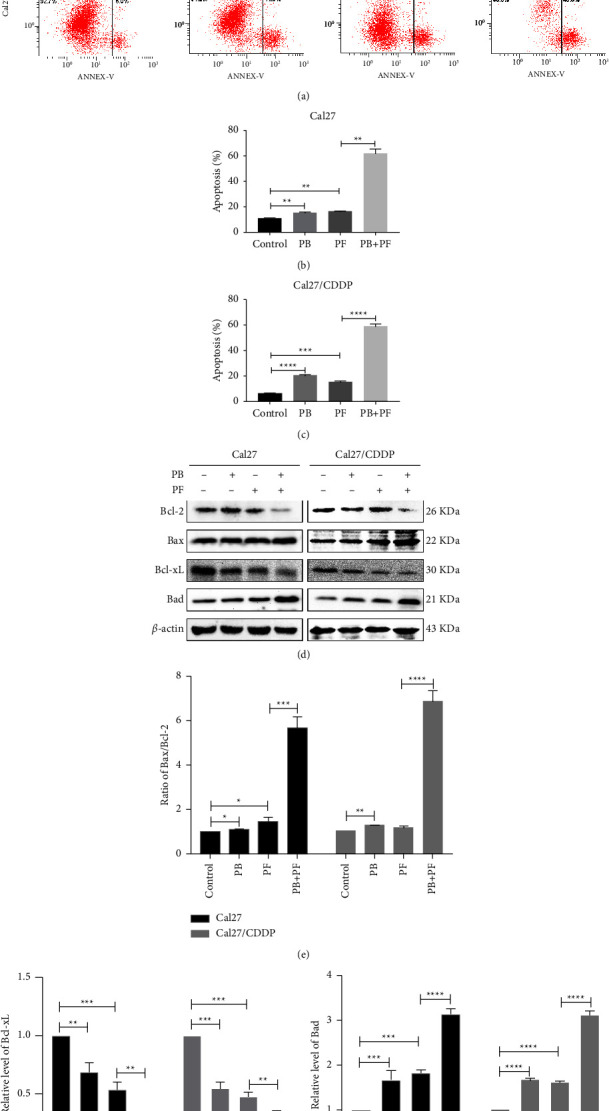
PB enhanced the proapoptotic effects of PF in TSCC cells. (a) Results of the flow cytometry assay demonstrated apoptosis in Cal27 and Cal27/CDDP cells following the treatment with PB and/or PF. (b-c) A histogram of the levels of apoptosis in Cal27 and Cal27/CDDP cells. (d) Representative blots of the proapoptotic protein Bax and Bad and antiapoptotic protein Bcl-2 and Bcl-xL in Cal27 and Cal27/CDDP cells after the treatment of PB and/or PF. (e) Ratio of Bax/Bcl-2 in Cal27 and Cal27/CDDP cells. (f-g) Histogram of the expression levels of Bcl-xL and Bad. Independent biological experiments were repeated three times, and statistical significance is denoted by ^*∗*^*P* <  0.05, ^*∗∗*^*P* < 0.01, ^*∗∗∗*^*P* < 0.001 and ^*∗∗∗∗*^*P* < 0.0001. PB, plumbagin; PF, cisplatin plus 5-fluorouracil; and TSCC, tongue squamous cell carcinoma.

Subsequently, the present study aimed to determine whether PB affected cellular apoptosis and whether apoptosis is a mechanism of synergism between PB and PF. Cal27 and Cal27/CDDP cells were treated with 2.5 *μ*M PB, PF (320 *μ*g/ml 5-Fu and 2.5 *μ*g/ml CDDP), and PB + PF for 24 h. Annexin V-FITC/PI flow cytometry was used to quantify apoptosis. As shown in Figure 5(a), PB and PF in combination significantly induced apoptosis. Compared with PF alone, PB + PF increased cellular apoptosis (both early and late apoptosis) by 2.88-fold in Cal27 cells (Figure 5(b)) and 2.93-fold in Cal27/CDDP cells (Figure 5(c)). The enhanced proapoptosis effect of PF by PB in Cal27/CDDP cells was more potent than that in Cal27 cells. Subsequently, western blot was carried out to explore the potential mechanisms underlying the proapoptotic effects of PB and PF in combination. The expression levels of apoptosis-related proteins, Bax, Bad, Bcl-2, and Bcl-xL, were examined in Cal27 and Cal27/CDDP cells. As shown in Figures 5(d)–5(g), the combined treatment of PB and PF significantly decreased the expression of the antiapoptotic protein Bcl-2 and Bcl-xL while increasing the expression of the proapoptotic proteins, Bax and Bad, in both cell lines, compared with PB or PF treatment alone. Collectively, these results indicated that PB enhanced the apoptosis-inducing effect of PF in TSCC cells.

### 3.4. Combined PB and PF Inhibit the PI3K/AKT/mTOR/p70S6K Pathway in TSCC Cells

**Figure 6:**
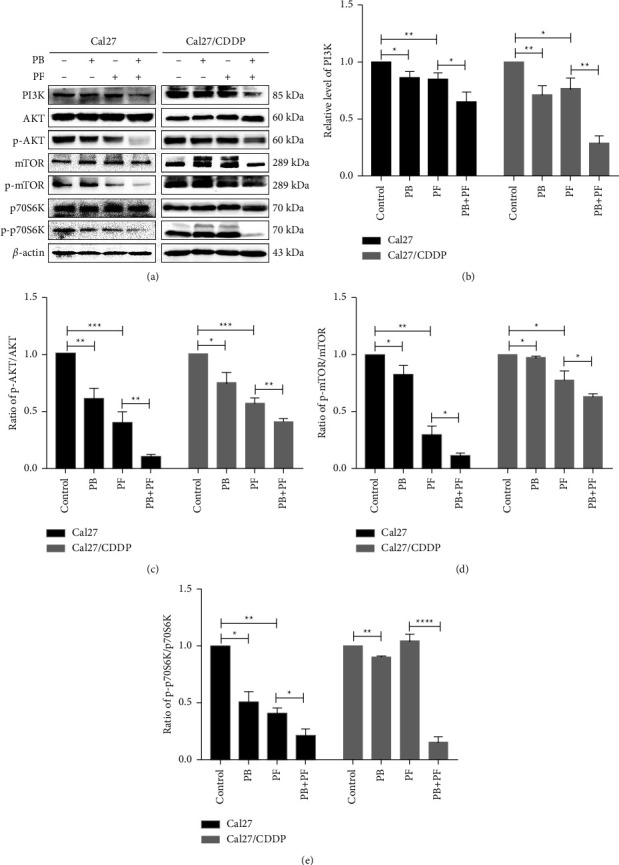
Combined PB and PF inhibited the PI3K/AKT/mTOR/p70S6K pathway in TSCC cells. (a) Representative blots demonstrating the expression of proteins involved in the PI3K/AKT/mTOR pathway in Cal27 and Cal27/CDDP cells following treatment with PB and/or PF. (b–e) A histogram demonstrating the expression level of PI3K, p-AKT/AKT, p-mTOR/mTOR, and p-p70S6K/p70S6K. Independent biological experiments were repeated three times. ^*∗*^*P* <  0.05, ^*∗∗*^*P* < 0.01, ^*∗∗∗*^*P* < 0.001, and ^*∗∗∗∗*^*P* < 0.0001. PB, plumbagin; PF, cisplatin plus 5-fluorouracil; TSCC, tongue squamous cell carcinoma; and p-, phosphorylated.

To further explore the molecular mechanisms underlying PB-enhanced PF chemotherapy in the inhibition of TSCC cells, western blot analysis was carried out to detect the expression of proteins involved in the PI3K/AKT/mTOR/p70S6K signaling pathway, which plays a key role in cell cycle and apoptosis. Compared with the control group, the protein expression levels of AKT, mTOR, and p70S6K in Cal27 and Cal27/CDDP cells in the PB group were not significantly altered. However, the protein expression levels of PI3K, p-AKT, p-mTOR, and p-p70S6K were all decreased. Compared with the PF group, the expression levels of PI3K, p-AKT, p-mTOR, and p-p70S6K in the cells of the PB + PF group were also decreased (Figures 6(a)–6(e)). These results indicated that a combination of PB and PF chemotherapy might inhibit the PI3K/AKT/mTOR/p70S6K signaling pathway in TSCC cells.

### 3.5. Combined PB and PF Inhibits TSCC Xenograft Tumor Growth *In Vivo*

**Figure 7:**
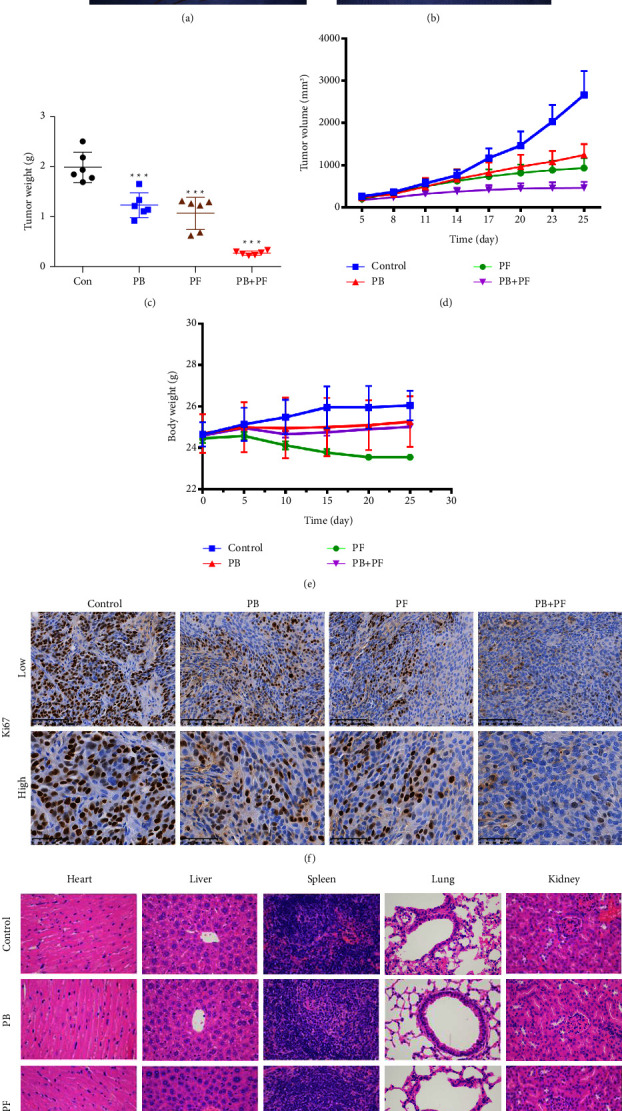
Combined treatment with PB and PF inhibited TSCC xenograft tumor growth *in vivo*. (a-b) Cal27/CDDP-derived xenograft models via subcutaneous injection into BALB/c nude mice. (c) Tumor weights in the PB + PF group were significantly lower than those in the control group. (d) Tumor volumes in the PB + PF group were significantly lower than those in the control group. (e) No significant changes in body weight were observed in the PB + PF group compared with the control group. (f) Ki67 expression in the PB + PF group was significantly lower than that in the control group. (g) H and E staining verified that the PB + PF group did not induce toxicity in major organs (*n* = 6/group). PB, plumbagin; PF, cisplatin plus 5-fluorouracil; and TSCC, tongue squamous cell carcinoma.

Based on the synergistic inhibition of Cal27 and Cal27/CDDP cells following treatment PB + PF, the present study aimed to determine whether similar therapeutic effects occurred in a subcutaneous cisplatin-resistant xenograft nude mice model. Cal27/CDDP cells were injected into the right flank of male nude mice. Results of the present study demonstrated that after 21 days of treatment, tumor growth was inhibited in PB or PF or PB + PF groups. Notably, PB + PF exerted the greatest inhibition of tumor volume and weight, compared with either PB or PF treatment alone (Figures 7(a)–7(d)). In addition, the weights of nude mice were significantly decreased following the treatment with PF, compared with either PB or PF treatment alone. On the other hand, PB + PF treatment reversed this phenomenon (Figure 7(e)). These results indicated that the combined treatment of PB + PF was more effective in tumor control and exerted a lower level of toxicity. In addition, H and E staining verified that PB + PF did not cause toxicity in the major organs, including the heart, liver, spleen, lung, and kidney (Figure 7(g)). Results of the present study also demonstrated that PB + PF significantly inhibited Ki67 expression, compared with PF alone (Figure 7(f)). Collectively, these results demonstrated that PB combined with PF might exhibit potential as a strategy to reduce PF resistance in TSCC while also reducing any systematic side effects.

## 4. Discussion

At present, neoadjuvant chemotherapy (NACT), systemic chemotherapy prior to surgery, or radiotherapy is used to meet the conditions required for subsequent surgery and radiotherapy to reduce tumor volumes and inhibit metastatic cells [21]. NACT is mainly used in patients with high pathological stage cancer and large tumor volumes. Thus, patients who were previously inoperable may be offered surgery in order to inhibit potential metastases. Notably, NACT is widely used in a variety of malignant tumors including TSCC. The currently used NACT regimens include the DBP regimen (pingyangmycin + cisplatin + 5-Fu), the PDF regimen (paclitaxel + cisplatin + 5-Fu), the TPF regimen (docetaxel + cisplatin + 5-Fu), and the PF scheme (cisplatin + 5-Fu), of which PF chemotherapy is regarded as the standard chemotherapy regimen for the treatment of TSCC [22]. However, the toxicity and adverse side effects of chemotherapeutic drugs and the decreased sensitivity of tumor cells to chemotherapeutic drugs greatly affect the efficacy. Therefore, a novel treatment regimen with low toxicity is required to enhance the sensitivity of TSCC to PF.

In recent years, more and more researchers have tended to develop natural herbal extracts due to their fewer adverse side effects and low costs [23]. PB is a kind of quinone extracted from the root of *Plumbago zeylanica L*., with antiatherosclerosis, anti-inflammatory, antibacterial, and antitumor pharmacological activity [11]. In recent years, more and more studies have shown that PB has an excellent killing effect on lung cancer, liver cancer, breast cancer, prostate cancer, and other malignant tumors [24]. Results of the present study demonstrated that PB combined with PF chemotherapy inhibited the proliferation, invasion, and migration of Cal27 and Cal27/CDDP cells. Results of *in vivo* assays demonstrated that the tumor weight and volume in the combined PB and PF group were reduced compared with cells in the group treated with PF alone. Furthermore, H and E staining verified that the combined treatment regimen did not cause toxicity in major organs, including the heart, liver, spleen, lung, and kidney. These results demonstrated that PB enhanced the therapeutic effect of PF while reducing its toxicity.

The reported antitumor mechanisms of PB include the elevation of cellular ROS, promotion of apoptosis, cell cycle arrest, and the inhibition of epithelial-mesenchymal transition [25]. Results of the present study demonstrated that PB combined with PF significantly inhibited the proliferation of Cal27 and Cal27/CDDP cells by inducing cell cycle arrest in the S phase and promoting cell apoptosis, compared with PF treatment alone. Apoptosis is a type of programmed cell death that can be classified into intrinsic apoptosis and extrinsic apoptosis. The mitochondrial pathway is the main regulatory pathway of intrinsic apoptosis [26]. The Bcl-2 family plays a central role in regulating mitochondrial function and outer membrane permeability. Notably, the Bcl-2 family of proteins can be divided into antiapoptotic and proapoptotic subfamilies [27]. The combined treatment of PB and PF significantly decreased the expression of antiapoptotic proteins Bcl-2 and Bcl-xL and increased the expression of proapoptotic proteins Bax and Bad in Cal27 and Cal27/CDDP cells, compared with PB or PF treatment alone. Collectively, these results indicated that PB enhanced the intrinsic apoptosis-inducing effect of PF in TSCC cells.

The PI3K/AKT/mTOR/p70S6K signaling pathway plays an important role in the occurrence and development of tumors. Jhou et al. [28] reported that chlorpromazine induced G_2_/M arrest and apoptosis via inhibiting the PI3K/AKT/mTOR pathway in oral cancer. Results of a previous study demonstrated that activation of the PI3K/AKT/mTOR/p70S6K signaling pathway is closely associated with chemoresistance in tumors. Lu et al. [29] found that the noncoding RNA-regulator of reprogramming (ROR) enhanced the sensitivity of breast cancer cells to the chemotherapeutic drug tamoxifen through inhibition of the PI3K/AKT/mTOR signaling pathway. Li et al. [30] also demonstrated that the downregulation of the PI3K/AKT/p70S6K signaling pathway using Guajadial reversed the multidrug resistance of breast cancer cells. Results of the present study demonstrated that the expression levels of PI3K and the ratios of phosphorylated (p)-AKT/AKT, p-mTOR/mTOR, and p-p70S6K/p70S6K were all decreased in the PB + PF group, compared with either of the PF or PB groups. Future studies will focus on the use of quantum nano-related technology to achieve targeted and efficient drug delivery.

Collectively, the results of the present study demonstrated that PB synergistically enhanced the anticancer effect of PF chemotherapy. PB inhibited proliferation, invasion, and migration and induced apoptosis of TSCC cells by regulating the PI3K/AKT/mTOR/p70S6K signaling pathways. This, therefore, enhanced the chemosensitivity of TSCC to PF.

## Data Availability

The datasets used and/or analyzed during the current study are available from the corresponding author upon reasonable request.

## Supplementary Materials

Supplementary Materials CI values of the combination of PB and PF in Cal27 and Cal27/CDDP cells are determined using Chou and Talalay analysis. CI, combination index; PB, plumbagin; PF, cisplatin plus 5-fluorouracil.
