# Supplementary material for: Plumbagin Enhances the Anticancer Effects of PF Chemotherapy via Downregulation of the PI3K/AKT/mTOR/p70S6K Pathway in Human Tongue Squamous Cell Carcinoma
Source: J Oncol. 2023 Feb 13;2023:8306514. doi: 10.1155/2023/8306514 (PMC9940972; doi:10.1155/2023/8306514)
Supplement: Supplementary Materials — CI values of the combination of PB and PF in Cal27 and Cal27/CDDP cells are determined using Chou and Talalay analysis. CI, combination index; PB, plumbagin; PF, cisplatin plus 5-fluorouracil. [file 8306514.f1.docx]

|  | Cal27 | | | | | Cal27/CDDP | | | | |
| --- | --- | --- | --- | --- | --- | --- | --- | --- | --- | --- |
| No. | PB  (μmol/l) | 5-Fu  (μg/ml) | CDDP  (μg/ml) | FA | CI | PB  (μmol/l) | 5-Fu  (μg/ml) | CDDP  (μg/ml) | FA | CI |
| 1 | 0.625 | 80 | 0.625 | 0.13 | 0.75069 | 0.625 | 80 | 0.625 | 0.11 | 0.79564 |
| 2 | 1.25 | 160 | 1.25 | 0.37 | 0.69857 | 1.25 | 160 | 1.25 | 0.25 | 0.90960 |
| 3 | 2.5 | 320 | 2.5 | 0.66 | 0.73550 | 2.5 | 320 | 2.5 | 0.60 | 0.82662 |
| 4 | 5 | 640 | 5 | 0.83 | 0.90989 | 5 | 640 | 5 | 0.90 | 0.70346 |
| 5 | 10 | 1280 | 10 | 0.96 | 0.81383 | 10 | 1280 | 10 | 0.98 | 0.67065 |

Table S1. The CI values of PB、CDDP and 5-Fu were calculated for the two TSCC cell lines
